# A dual investigation of the effect of dietary supplementation with licorice flavonoid oil on anthropometric and biochemical markers of health and adiposity

**DOI:** 10.1186/1476-511X-10-29

**Published:** 2011-02-10

**Authors:** Zach W Bell, Robert E Canale, Richard J Bloomer

**Affiliations:** 1 Cardiorespiratory/Metabolic Laboratory, The University of Memphis, Memphis, TN 38152, USA

## Abstract

**Background:**

Licorice flavonoid oil (LFO) has been reported to minimize visceral adipose tissue gain in obese mice and to result in a decrease in body weight and body fat in humans; the effects of which may be more pronounced when administered in an overfed state.

**Methods:**

We investigated the effects of LFO in two separate studies. Study 1 included a sample of overweight or grade I-II obese men and women (N = 22) who followed their usual dietary and physical activity programs. Study 2 included a sample of athletic men who followed their usual dietary and physical activity programs but consumed a daily supplemental meal (25% above daily energy requirements) in an attempt to induce a state of overfeeding. In both studies, subjects were randomly assigned (double-blind) to either LFO or a placebo for eight weeks, and anthropometric and multiple biochemical outcomes (e.g., markers of oxidative stress, markers of insulin sensitivity, blood lipids, etc.) were obtained before and following the intervention.

**Results:**

No differences of statistical significance were noted between LFO and placebo for any measured variable in Study 1 or Study 2. When investigating the percent change from baseline for data in Study 2, although not of statistical significance, subjects in the LFO condition experienced less overall fat gain, as well as attenuation in the elevation in selected blood lipids (e.g., cholesterol, LDL-C, and triglycerides).

**Conclusion:**

These combined data indicate little effect of LFO supplementation within a sample of overweight/obese men and women or athletic men, with the possible exception of attenuation in body fat gain and selected components of the blood lipid panel in response to an overfeeding condition.

## Background

Licorice flavonoid oil (LFO) has been reported to minimize body weight and visceral adipose tissue gain in obese mice [1,2], and to result in a decrease in body weight and body fat in humans [3]. However, human studies to date have focused primarily on anthropometric outcomes (e.g., abdominal fat assessment via computed tomography [CT] scans), while also including measures of blood glucose and lipids. No human study has included other important biomarkers of metabolic and cardiovascular health such as adiponectin, resistin, C-reactive protein, and measures of oxidative stress and antioxidant capacity.

Licorice flavonoid oil has been studied for its mechanistic actions associated with reduction of abdominal fat [4]. Specifically, LFO has been shown to possess nutrigenomic properties that effect cellular pathways with regards to decreasing the mRNA levels of rate-limiting enzymes involved in the hepatic fatty acid synthetic pathway, while increasing the mRNA levels of a rate-limiting enzyme in the hepatic fatty acid oxidative pathway. Honda and colleagues suggest that a decrease in abdominal adipose tissue weight may be mediated by the transcriptional regulation of SREBP-1c and PPAR-alpha in the liver [4]. Aside from the potential effects of LFO on adipose tissue accumulation, a flavonoid component of the licorice root, glabridin, has been shown to have antioxidant properties [5], which may assist in attenuating lipid peroxidation.

DNA microarray analysis indicates that the effects of LFO are specific to the genes involved in the regulation of lipid metabolism [1], and that the effects may be most pronounced when the supplement is taken with a meal; in particular a meal rich in dietary fat. Therefore, it is possible that LFO supplementation during periods of overfeeding may be helpful in attenuating the typical gain in body weight/fat, as well as the rise in circulating blood lipids.

Considering the above, we undertook two separate pilot studies to investigate the effect of LFO supplementation on anthropometric and biochemical markers of health and adiposity. The first study included recreationally active men and women who were overweight or grade I-II obese (body mass index (BMI): 25.0-36.0 kg·m^-2^) and followed their usual dietary and physical activity programs. The second study included athletic (i.e., resistance trained) men in very good physical condition, who also followed their usual dietary and physical activity programs but consumed a daily supplemental meal in an attempt to induce an acute state of overfeeding. In both studies, subjects were randomly assigned to either LFO or a placebo for a period of eight weeks, and outcome measures were obtained before and following the intervention.

## Methods and Procedures

### Subjects

For Study 1, 22 recreationally active men or women (2-4 days per week of moderate exercise) between the ages of 20 and 53 years participated. Subjects were overweight or grade I-II obese based on BMI classification. For Study 2, 23 athletic men between the ages of 19 and 35 years participated. For both studies, subjects were nonsmokers and did not have any cardiovascular, metabolic, or orthopedic problems that might affect their ability to perform exercise. Health history, drug and dietary supplement usage, and physical activity questionnaires were completed by all subjects to determine eligibility. Prior to participation, each subject was informed of all procedures, potential risks, and benefits associated with the study through both verbal and written form in accordance with the procedures approved by the University Institutional Review Board for Human Subjects Research. All subjects signed an informed consent form prior be being admitted.

### Screening

During the initial visit to the laboratory, subjects completed the informed consent form, health and physical activity questionnaires. Subjects were provided with food logs and instructions regarding how to complete these logs during the week prior to beginning their assigned condition and the final week of the assigned condition (described below). Subjects also received a detailed schedule for the entire study period mapping out all pertinent dates of participation.

### Testing

For the two visits described below, subjects reported to the laboratory in the morning hours (5:00-10:00 am) following a 10 hour overnight fast. After resting for 10 minutes, subjects' heart rate and blood pressure was measured, and a blood sample was obtained. Following this, subjects' height, weight, waist and hip circumference, skinfold thickness (7 site), and body composition was measured. Body composition was determined by dual energy x-ray absorptiometry (DEXA; Hologic QDR-4500W) using a 4-minute fan array. Specifically, total and regional (trunk specific) body fat were determined. The assessment was performed by a licensed technician. These exact procedures were followed for both test days.

### Blood Sampling and Biochemistry

Blood was collected from subjects on two different days throughout the course of the study: pre intervention (day 1 of the study) and post intervention (day 57 of the study). On each occasion, venous blood samples (25 mL; 5 teaspoons) were taken from subjects via needle and Vacutainer^®^. Blood samples were collected in a fasted and 10 minute rested state. Following collection, samples were processed and immediately placed in the refrigerator or the freezer, depending on the sample. A portion of blood samples were sent to Laboratory Corporation of America (LabCorp) for analysis of complete blood count, comprehensive metabolic panel, and lipid panel. The complete blood count was determined using an automated cell counter (Coulter LH750). The comprehensive metabolic panel was determined using automated procedures (Roche/Hitachi Modular). The lipid panel was determined using enzymatic procedures (Roche/Hitachi Modular). For Study 1 only, insulin was determined by LabCorp using an immuno-chemiluminescent assay procedure (Roche Modular E170) and C-reactive protein was determined using a high-sensitivity, particle-enhanced turbidimetric immunoassay (Roche Integra 800). The homeostasis model assessment (HOMA-IR) was used as an index of insulin resistance [6] and calculated as: [fasting glucose (mg·dL^-1^) × fasting insulin (μU·mL^-1^)]/405.

All remaining blood was stored at -70°C until analyzed for outcome variables for Study 1 (resistin, adiponectin, free fatty acids, malondialdehyde, hydrogen peroxide, and Trolox Equivalent Antioxidant Capacity) and Study 2 (free fatty acids, malondialdehyde, insulin). Both resistin and adiponectin were determined in serum using an enzyme immunoassay (EIA) following the instructions of the manufacturer (SPI-Bio, Berin Pharma, France). Free fatty acids were determined in plasma using the Free Fatty Acid Quantification Kit following the instructions of the manufacturer (BioVision). Malondialdehyde was determined in plasma following the procedures of Jentzsch et al [7], using reagents purchased from Northwest Life Science Specialties (Vancouver, WA). Hydrogen peroxide was determined in plasma using the Amplex Red reagent method as described by the manufacturer (Molecular Probes, Invitrogen Detection Technologies, Eugene, OR). Antioxidant capacity was determined in serum using procedures outlined by the reagent provided (Sigma Chemical, St. Louis, MO). Insulin samples for Study 2 were determined in serum using a solid phase enzyme linked immunosorbent assay (ELISA) kit following the instructions of the manufacturer (Calbiotech, Spring Valley, CA).

### Independent Variable (Condition)

For both studies, subjects were randomly assigned in a double-blind manner to consume either a placebo or LFO at a dosage at 300 mg·day^-1 ^(taken in 3 capsules [100 mg each] with the evening meal. Kaneka Glavonoid™ is a licorice derived (Glycyrrhiza glabra L.) flavonoid (Licorice Glabra Polyphenol) oil and used as LFO in the present study. Glavonoid™ has been reported to have high bioavailability [8] and to be safe in animal toxicology studies, with no genotoxicity or carcinogenicity observed. Furthermore, no clinically significant adverse effects have been noted in humans with dosing up to 400 mg/d (delivered as 1200 mg/d of LFO) [9] and 600 mg/d (delivered as 1800 mg/d of LFO) [10] for four weeks. The particular flavonoids used in Glavonoid™ are specific to the Asian licorice glabra species; hence, results from other studies focused on LFO may not be directly comparable. Supposedly, this particular Asian licorice specie has higher levels of glabridin, compared to other forms of LFO, possibly due to the use of a double extraction method used in the processing of Kaneka Glavonoid™. Glavonoid™ is standardized to 30% licorice glabra polyphenol and 3% glabridin. The placebo (medium chain triglycerides and bees wax) and Glavonoid™ capsules were identical in appearance and were dispensed to subjects in unlabeled bottles. Capsule counts upon bottle return determined compliance. Subjects received their assigned condition for a period of eight (8) weeks.

### Dietary Records and Physical Activity

For both studies, subjects were instructed to maintain their normal diet during the entire study period and to record intake during the week prior to each test day. The exception was the addition of the supplemental meal used in Study 2 (described below). Diet records were analyzed for total calories, protein, carbohydrate, fat, and a variety of micronutrients (Food Processor SQL, version 9.9, ESHA Research, Salem, OR). Subjects were asked to maintain their normal physical activity habits during the study period, with the exception of the two days (48 hours) prior to each test day, in which they were asked not to perform any strenuous exercise.

### Supplemental Meal (Study 2 only)

In an attempt to induce an acute state of overfeeding and body weight/fat gain, subjects in Study 2 were required to consume a supplemental meal each day of the intervention period. This meal was consumed prior to bedtime (i.e., between the final daily meal and bedtime), along with the Glavonoid™ or placebo capsules. The meal consisted of a combination of whole milk and a meal replacement powder (Muscle Juice™ 2544; Ultimate Nutrition). All subjects consumed two cups of whole milk (300 kilocalories), and the dosage of meal replacement powder was based on subjects' body weight and equal to a combined kilocalorie load of 8 kilocalories per kilogram (milk + powder). For example, a 180 pound man (82 kg) consumed an additional 656 calories in the form of this supplemental meal/shake (300 kilocalories of milk + 356 kilocalories of powder). For most subjects, this provided a kilocalorie load of approximately 25% of their daily intake. This meal was comprised of approximately 30% fat, 20% protein, and 50% carbohydrate. Subjects were provided with adequate meal replacement powder every four weeks and were instructed to consume the supplemental meal in addition to their regular daily food and beverage intake.

### Statistical Analysis

For both studies, outcome measures were analyzed using a 2 (condition) × 2 (pre/post intervention) analysis of variance (ANOVA). The data are presented as mean ± SEM. All analyses were performed using JMP statistical software (version 4.0.3, SAS Institute, Cary, NC). Statistical significance was set at P ≤ 0.05.

## Results

Subject compliance to treatment, descriptive characteristics, anthropometric and hemodynamic data are presented in Table 1 (Study 1) and Table 2 (Study 2). In both studies, compliance to treatment was excellent and not different between conditions. For Study 1, no significant interactions or main effects were noted (p > 0.05). For Study 2, a condition effect was noted for age (p = 0.04), DEXA total body fat (p = 0.03), DEXA trunk body fat (p = 0.05), and total fat mass (p = 0.02). No other significant effects were noted (p > 0.05).

**Table 1:** Descriptive characteristics, anthropometric and hemodynamic data for 22 men and women assigned to Glavonoid™ or placebo for eight weeks.

Variable	Glavonoid™ Pre (n = 11)	Glavonoid™ Post (n = 11)	Placebo Pre (n = 11)	Placebo Post (n = 11)
Compliance to Treatment (%)	NA	94.8 ± 1.4	NA	97.0 ± 1.2
Age (years)	28.4 ± 2.8	28.4 ± 2.8	25.7 ± 1.8	25.7 ± 1.8
Height (cm)	173.9 ± 3.5	173.9 ± 3.5	175.4 ± 2.8	175.4 ± 2.8
Body Weight (kg)	88.7 ± 4.0	88.6 ± 3.7	92.3 ± 2.9	92.0 ± 3.0
BMI (kg·m^-2^)	29.4 ± 1.3	29.4 ± 1.2	30.2 ± 1.2	30.1 ± 1.3
Waist (cm)	88.5 ± 3.2	88.9 ± 3.0	90.7 ± 2.0	90.9 ± 2.1
Hip (cm)	113.8 ± 2.3	114.2 ± 2.3	114.6 ± 2.3	113.7 ± 2.2
Waist:Hip	0.78 ± 0.02	0.78 ± 0.02	0.79 ± 0.02	0.80 ± 0.02
DEXA Total Body Fat (%)	33.0 ± 2.9	32.7 ± 3.2	32.1 ± 2.1	32.2 ± 2.1
DEXA Trunk Body Fat (%)	34.1 ± 2.7	34.0 ± 3.2	32.8 ± 2.4	32.5 ± 2.2
Total Fat Mass (kg)	29.1 ± 2.8	28.8 ± 2.9	29.8 ± 2.4	29.7 ± 2.4
Total Fat Free Mass (kg)	59.7 ± 3.9	59.8 ± 3.9	62.5 ± 2.5	62.2 ± 2.5
Skinfold Thickness (mm)	228.7 ± 19.0	221.0 ± 16.6	231.6 ± 11.1	217.9 ± 13.2
Heart Rate (bpm)	64.8 ± 3.1	63.9 ± 2.2	66.4 ± 1.8	68.1 ± 2.7
Systolic Blood Pressure (mmHg)	114.7 ± 3.4	114.4 ± 2.8	108.9 ± 3.4	111.8 ± 2.4
Diastolic Blood Pressure (mmHg)	75.1 ± 3.2	74.9 ± 3.0	70.3 ± 2.1	73.3 ± 2.5

Values are mean ± SEM.

No condition × pre/post intervention interaction effects noted (p > 0.05).

No condition effects noted (p > 0.05).

No pre/post intervention effects noted (p > 0.05).

**Table 2:** Descriptive characteristics, anthropometric and hemodynamic data for 23 resistance trained men assigned to Glavonoid™ or placebo for eight weeks.

Variable	Glavonoid™ Pre (n = 12)	Glavonoid™ Post (n = 12)	Placebo Pre (n = 11)	Placebo Post (n = 11)
Resistance Training (years)	4.3 ± 0.9	NA	5.1 ± 0.9	NA
Compliance to Treatment (%)	NA	96.1 ± 1.4	NA	96.3 ± 1.0
Age (years)*	24.2 ± 1.0	24.3 ± 1.0	21.8 ± 1.3	21.9 ± 1.3
Height (cm)	178.2 ± 1.7	178.2 ± 1.7	177.4 ± 1.6	177.4 ± 1.6
Body Weight (kg)	76.2 ± 2.7	78.1 ± 2.8	75.1 ± 2.6	76.7 ± 2.7
BMI (kg·m^-2^)	23.9 ± 0.6	24.5 ± 0.7	23.8 ± 0.6	24.3 ± 0.6
Waist (cm)	79.8 ± 1.2	80.8 ± 1.2	79.0 ± 1.5	80.1 ± 1.6
Hip (cm)	97.6 ± 1.5	98.2 ± 1.5	97.6 ± 1.3	97.2 ± 1.4
Waist:Hip	0.82 ± 0.0	0.82 ± 0.0	0.81 ± 0.0	0.82 ± 0.0
DEXA Total Body Fat (%)*	14.6 ± 1.1	14.9 ± 1.2	11.9 ± 1.0	12.8 ± 1.0
DEXA Trunk Body Fat (%)*	14.5 ± 1.1	14.9 ± 1.2	11.9 ± 1.1	12.9 ± 1.1
Total Fat Mass (kg)*	11.0 ± 0.7	11.5 ± 0.8	8.9 ± 0.8	9.8 ± 0.8
Total Fat Free Mass (kg)	65.1 ± 2.8	66.5 ± 2.9	66.1 ± 2.5	67.0 ± 2.6
Skinfold Thickness (mm)	82.8 ± 4.3	91.9 ± 5.7	71.9 ± 6.5	85.6 ± 8.0
Heart Rate (bpm)	62.2 ± 2.5	62.1 ± 2.4	59.9 ± 3.0	56.2 ± 3.6
Systolic Blood Pressure (mmHg)	125.1 ± 3.0	126.8 ± 2.2	122.8 ± 2.4	125.0 ± 3.4
Diastolic Blood Pressure (mmHg)	74.1 ± 3.3	75.2 ± 3.3	69.2 ± 3.7	72.5 ± 3.1

Values are mean ± SEM.

No condition × pre/post intervention interaction effects noted (p > 0.05).

* Condition effect noted for age (p = 0.04), DEXA Total Body Fat (p = 0.03), DEXA Trunk Body Fat (p = 0.05), and Total Fat Mass (p = 0.02). No other condition effects noted (p > 0.05).

No pre/post intervention effects noted (p > 0.05).

Complete blood count data are presented in Table 3 (Study 1) and Table 4 (Study 2). For Study 1, no significant interactions or main effects were noted (p > 0.05). For Study 2, pre/post intervention effects were noted for hematocrit (p = 0.03) and MCHC (p = 0.005). No other significant effects were noted (p > 0.05).

**Table 3:** Complete blood count data for 22 men and women assigned to Glavonoid™ or placebo for eight weeks.

Variable	Glavonoid™ Pre (n = 11)	Glavonoid™ Post (n = 11)	Placebo Pre (n = 11)	Placebo Post (n = 11)
WBC (10^3^· μL^-1^)	5.8 ± 0.4	5.8 ± 0.4	6.6 ± 0.5	6.0 ± 0.6
RBC (10^6^· μL^-1^)	4.5 ± 0.1	4.4 ± 0.1	4.4 ± 0.1	4.5 ± 0.1
Hemoglobin (g·dL^-1^)	13.8 ± 0.4	13.6 ± 0.3	13.5 ± 0.5	14.0 ± 0.5
Hematocrit (%)	39.7 ± 1.0	40.1 ± 1.0	39.2 ± 1.4	40.9 ± 1.4
MCV (fL)	88.7 ± 1.1	90.6 ± 1.4	90.1 ± 1.1	90.9 ± 1.6
MCH (pg)	30.7 ± 0.4	30.7 ± 0.4	31.0 ± 0.6	31.1 ± 0.5
MCHC (g·dL^-1^)	34.7 ± 0.2	34.0 ± 0.2	34.5 ± 0.3	34.2 ± 0.2
RDW (%)	13.4 ± 0.2	13.5 ± 0.3	13.8 ± 0.4	13.5 ± 0.4
Platelets (10^3^· μL^-1^)	201.7 ± 14.2	200.9 ± 14.8	212.6 ± 20.0	215.5 ± 18.8
Neutrophils (%)	55.4 ± 1.9	58.3 ± 4.1	52.2 ± 3.0	51.2 ± 3.3
Lymphocytes (%)	33.5 ± 2.0	31.2 ± 3.6	37.4 ± 2.9	37.6 ± 3.0
Monocytes (%)	7.5 ± 0.5	6.9 ± 0.6	6.9 ± 0.8	7.3 ± 0.7
Eosinophils (%)	3.0 ± 0.6	3.1 ± 0.5	3.3 ± 0.8	3.4 ± 0.7
Basophils (%)	0.6 ± 0.2	0.5 ± 0.2	0.2 ± 0.1	0.5 ± 0.2

Values are mean ± SEM.

No condition × pre/post intervention interaction effects noted (p > 0.05).

No condition effects noted (p > 0.05).

No pre/post intervention effects noted (p > 0.05).

**Table 4:** Complete blood count data for 23 resistance trained men assigned to Glavonoid™ or placebo for eight weeks.

Variable	Glavonoid™ Pre (n = 12)	Glavonoid™ Post (n = 12)	Placebo Pre (n = 11)	Placebo Post (n = 11)
WBC (10^3^· μL^-1^)	6.7 ± 0.4	6.4 ± 0.5	6.5 ± 0.6	6.1 ± 0.4
RBC (10^6^· μL^-1^)	4.8 ± 0.8	4.8 ± 0.1	4.8 ± 0.1	4.9 ± 0.1
Hemoglobin (g·dL^-1^)	14.9 ± 0.2	14.9 ± 0.3	15.0 ± 0.3	15.3 ± 0.2
Hematocrit (%)**	43.1 ± 0.5	44.0 ± 0.7	43.2 ± 0.7	45.3 ± 0.7
MCV (fL)	90.7 ± 1.0	91.7 ± 1.2	90.5 ± 1.1	92.2 ± 1.3
MCH (pg)	31.2 ± 0.4	31.0 ± 0.4	31.3 ± 0.4	31.2 ± 0.5
MCHC (g·dL^-1^)**	34.4 ± 0.2	33.9 ± 0.2	34.6 ± 0.2	33.9 ± 0.2
RDW (%)	12.9 ± 0.2	13.0 ± 0.1	13.0 ± 0.1	13.2 ± 0.2
Platelets (10^3^· μL^-1^)	208.3 ± 14.1	199.5 ± 10.4	206.8 ± 13.1	206.0 ± 12.9
Neutrophils (%)	57.8 ± 2.6	56.6 ± 2.9	57.8 ± 2.8	58.1 ± 2.5
Lymphocytes (%)	31.4 ± 2.4	33.0 ± 2.6	30.8 ± 2.6	30.7 ± 2.2
Monocytes (%)	7.9 ± 0.4	7.6 ± 0.5	8.5 ± 0.6	7.3 ± 0.6
Eosinophils (%)	2.5 ± 0.4	2.8 ± 0.4	2.5 ± 0.3	3.4 ± 0.8
Basophils (%)	0.3 ± 0.1	0.3 ± 0.0	0.4 ± 0.2	0.5 ± 0.2

Values are mean ± SEM.

No condition × pre/post intervention interaction effects noted (p > 0.05).

No condition effects noted (p > 0.05).

** Pre/post intervention effect noted for Hematocrit (p = 0.03) and MCHC (p = 0.005). No other pre/post intervention effects noted (p > 0.05).

Metabolic panel data are presented in Table 5 (Study 1) and Table 6 (Study 2). For Study 1, a condition × pre/post intervention interaction effect was noted for sodium (p = 0.04), a condition effect was noted for sodium (p = 0.04), and a pre/post intervention effect was noted for AST (SGOT) (p = 0.02). No other significant effects were noted (p > 0.05). For Study 2, a condition effect was noted for chloride (p = 0.04), Alk Phos (p = 0.003), and ALT (SGOT) (p = 0.04). A pre/post intervention effect was noted for CO_2 _(p = 0.01) and calcium (p = 0.007). No other significant effects were noted (p > 0.05).

**Table 5:** Metabolic panel data for 22 men and women assigned to Glavonoid™ or placebo for eight weeks.

Variable	Glavonoid™ Pre (n = 11)	Glavonoid™ Post (n = 11)	Placebo Pre (n = 11)	Placebo Post (n = 11)
Insulin (μU·mL^-1^)	7.9 ± 1.3	9.2 ± 1.5	9.1 ± 0.5	8.4 ± 1.2
HOMA-IR	1.6 ± 0.3	2.0 ± 0.4	1.9 ± 0.1	1.9 ± 0.3
Adiponectin (μg·mL^-1^)	17.8 ± 1.7	19.0 ± 2.7	15.3 ± 3.4	15.3 ± 3.2
Resistin (ng·mL^-1^)	5.7 ± 0.4	5.9 ± 0.6	5.1 ± 0.7	4.8 ± 0.5
Glucose (mg·dL^-1^)	84.4 ± 1.9	85.7 ± 2.9	83.4 ± 2.7	88.0 ± 4.2
BUN (mg·dL^-1^)	13.6 ± 1.2	12.6 ± 1.3	12.7 ± 1.0	13.8 ± 1.3
Creatinine (mg·dL^-1^)	1.0 ± 0.1	1.0 ± 0.1	0.9 ± 0.0	0.9 ± 0.0
BUN:Creatinine	14.2 ± 0.8	13.1 ± 0.6	14.3 ± 1.5	15.0 ± 1.6
Sodium (mmol·L^-1^)*†	137.5 ± 0.4	139.0 ± 0.5	138.9 ± 0.5	139.0 ± 0.5
Potassium (mmol·L^-1^)	4.1 ± 0.1	4.2 ± 0.1	4.3 ± 0.1	4.5 ± 0.2
Chloride (mmol·L^-1^)	102.5 ± 1.1	102.1 ± 0.7	102.7 ± 0.6	103.1 ± 0.5
CO_2 _(mmol·L^-1^)	24.0 ± 0.9	26.0 ± 0.8	24.3 ± 0.7	25.3 ± 0.7
Calcium (mg·dL^-1^)	9.4 ± 0.1	9.2 ± 0.1	9.4 ± 0.2	9.1 ± 0.1
Protein (g·dL^-1^)	6.9 ± 0.1	6.9 ± 0.1	6.9 ± 0.1	7.0 ± 0.1
Albumin (g·dL^-1^)	4.3 ± 0.1	4.3 ± 0.1	4.3 ± 0.1	4.3 ± 0.1
Globulin (g·dL^-1^)	2.6 ± 0.1	2.6 ± 0.1	2.6 ± 0.1	2.7 ± 0.1
A:G	1.7 ± 0.1	1.7 ± 0.1	1.7 ± 0.1	1.7 ± 0.1
Bilirubin (mg·dL^-1^)	0.5 ± 0.1	0.4 ± 0.1	0.6 ± 0.1	0.5 ± 0.1
Alk Phos (IU·L^-1^)	67.5 ± 6.8	65.3 ± 6.5	72.6 ± 6.3	70.9 ± 7.4
AST (SGOT) (IU·L^-1^)**	23.7 ± 2.5	26.7 ± 2.3	21.2 ± 2.1	19.9 ± 0.9
ALT (SGPT) (IU·L^-1^)	23.5 ± 4.0	28.9 ± 5.2	20.5 ± 3.9	21.2 ± 4.9

Values are mean ± SEM.

† Condition × pre/post intervention interaction effect noted for Sodium (p = 0.04). No other condition × pre/post intervention interaction effects noted (p > 0.05).

* Condition effect noted for Sodium (p = 0.04). No other condition effects noted (p > 0.05).

** Pre/post intervention effect noted for AST (SGOT) (p = 0.02). No other pre/post intervention effects noted (p > 0.05).

**Table 6:** Metabolic panel data for 23 resistance trained men assigned to Glavonoid™ or placebo for eight weeks.

Variable	Glavonoid™ Pre (n = 12)	Glavonoid™ Post (n = 12)	Placebo Pre (n = 11)	Placebo Post (n = 11)
Insulin (μU·mL^-1^)	3.4 ± 0.2	3.5 ± 0.2	3.4 ± 0.1	3.6 ± 0.2
HOMA-IR	0.7 ± 0.0	0.8 ± 0.0	0.7 ± 0.1	0.8 ± 0.1
Glucose (mg·dL^-1^)	88.5 ± 2.7	93.8 ± 1.3	87.3 ± 3.2	89.3 ± 2.1
BUN (mg·dL^-1^)	18.8 ± 1.6	18.3 ± 1.2	17.9 ± 1.2	19.4 ± 1.4
Creatinine (mg·dL^-1^)	1.2 ± 0.1	1.1 ± 0.0	1.1 ± 0.0	1.1 ± 0.0
BUN:Creatinine	16.4 ± 1.4	16.7 ± 0.9	16.4 ± 1.1	18.4 ± 1.4
Sodium (mmol·L^-1^)	140.1 ± 0.7	140.6 ± 0.8	139.7 ± 0.6	139.0 ± 1.0
Potassium (mmol·L^-1^)	4.4 ± 0.1	4.5 ± 0.1	4.3 ± 0.1	4.4 ± 0.1
Chloride (mmol·L^-1^)*	101.1 ± 0.7	101.2 ± 0.6	101.5 ± 0.5	99.5 ± 0.8
CO_2 _(mmol·L^-1^)**	24.6 ± 0.6	26.3 ± 0.4	25.5 ± 0.8	27.0 ± 0.5
Calcium (mg·dL^-1^)**	9.8 ± 0.1	9.5 ± 0.1	9.6 ± 0.1	9.5 ± 0.1
Protein (g·dL^-1^)	7.1 ± 0.1	7.1 ± 0.1	6.9 ± 0.1	7.2 ± 0.1
Albumin (g·dL^-1^)	4.5 ± 0.1	4.6 ± 0.1	4.4 ± 0.1	4.6 ± 0.1
Globulin (g·dL^-1^)	2.6 ± 0.1	2.6 ± 0.1	2.4 ± 0.1	2.6 ± 0.1
A:G	1.8 ± 0.1	1.8 ± 0.1	1.9 ± 0.1	1.8 ± 0.1
Bilirubin (mg·dL^-1^)	0.8 ± 0.2	0.6 ± 0.1	0.6 ± 0.1	0.7 ± 0.1
Alk Phos (IU·L^-1^)*	70.4 ± 5.0	71.2 ± 4.2	83.0 ± 3.3	84.5 ± 3.3
AST (SGOT) (IU·L^-1^)	25.8 ± 3.4	27.8 ± 3.1	30.0 ± 4.2	26.7 ± 2.1
ALT (SGPT) (IU·L^-1^)*	20.3 ± 1.9	21.0 ± 2.2	27.3 ± 2.5	26.7 ± 4.5

Values are mean ± SEM.

No significant condition × pre/post intervention interaction effects noted (p > 0.05).

* Condition effect noted for Chloride (p = 0.04), Alk Phos (p = 0.003), and ALT (SGOT) (p = 0.04). No other condition effects noted (p > 0.05).

** Pre/post intervention effect noted for CO_2 _(p = 0.01) and Calcium (p = 0.007). No other pre/post intervention effects noted (p > 0.05).

Note: Insulin and HOMA-IR data missing for one subject in Glavonoid™ condition (n = 11).

Lipid panel and free fatty acid data are presented in Table 7 (Study 1) and Table 8 (Study 2; in addition to malondialdehyde data). For Study 1, no significant interactions or main effects were noted (p > 0.05). For Study 2, a pre/post intervention effect was noted for cholesterol (p = 0.02) and LDL-C (p = 0.03). No other significant effects were noted (p > 0.05).

**Table 7:** Lipid panel data for 22 men and women assigned to Glavonoid™ or placebo for eight weeks.

Variable	Glavonoid™ Pre (n = 11)	Glavonoid™ Post (n = 11)	Placebo Pre (n = 11)	Placebo Post (n = 11)
Cholesterol (mg·dL^-1^)	184.5 ± 11.8	174.2 ± 10.2	158.4 ± 10.2	168.3 ± 12.9
Triglycerides (mg·dL^-1^)	113.8 ± 16.8	98.2 ± 17.8	79.3 ± 16.6	71.0 ± 17.3
HDL-C (mg·dL^-1^)	52.7 ± 3.1	52.8 ± 3.7	47.6 ± 6.3	48.0 ± 4.5
VLDL-C (mg·dL^-1^)	22.7 ± 3.3	19.6 ± 3.5	16.0 ± 3.3	14.1 ± 3.4
LDL-C (mg·dL^-1^)	109.1 ± 10.5	101.7 ± 9.2	94.8 ± 7.9	106.2 ± 11.0
LDL-C/HDL-C	2.2 ± 0.3	2.1 ± 0.3	2.3 ± 0.4	2.4 ± 0.4
Total:HDL-C	3.7 ± 0.4	3.5 ± 0.4	3.8 ± 0.5	3.8 ± 0.5
Free Fatty Acids (mmol·L^-1^)	0.33 ± 0.05	0.36 ± 0.04	0.30 ± 0.04	0.23 ± 0.04

Values are mean ± SEM.

No condition × pre/post intervention interaction effects noted (p > 0.05).

No condition effects noted (p > 0.05).

No pre/post intervention effects noted (p > 0.05).

**Table 8:** Lipid panel data for 23 resistance trained men assigned to Glavonoid™ or placebo for eight weeks.

Variable	Glavonoid™ Pre (n = 12)	Glavonoid™ Post (n = 12)	Placebo Pre (n = 11)	Placebo Post (n = 11)
Cholesterol (mg·dL^-1^)**	147.9 ± 6.4	159.8 ± 8.0	148.4 ± 7.0	178.7 ± 11.6
Triglycerides (mg·dL^-1^)	76.6 ± 10.4	81.3 ± 10.6	76.4 ± 10.7	95.8 ± 8.9
HDL-C (mg·dL^-1^)	48.0 ± 2.2	47.8 ± 2.4	47.9 ± 2.1	50.0 ± 2.7
VLDL-C (mg·dL^-1^)	15.4 ± 2.1	16.3 ± 2.2	15.3 ± 2.2	19.3 ± 1.8
LDL-C (mg·dL^-1^)**	84.5 ± 6.2	95.7 ± 8.1	85.2 ± 5.7	109.5 ± 10.0
LDL-C/HDL-C	1.8 ± 0.2	2.1 ± 0.2	1.8 ± 0.2	2.2 ± 0.2
Total:HDL-C	3.1 ± 0.2	3.5 ± 0.3	3.2 ± 0.2	3.6 ± 0.2
Free Fatty Acids (mmol·L^-1^)	0.41 ± 0.1	0.30 ± 0.0	0.30 ± 0.0	0.32 ± 0.1
Malondialdehyde (μmol·L^-1^)	0.88 ± 0.09	0.84 ± 0.08	0.87 ± 0.11	0.84 ± 0.09

Values are mean ± SEM.

No significant condition × pre/post intervention interaction effects noted (p > 0.05).

No condition effects noted (p > 0.05).

** Significant pre/post intervention effect noted for Cholesterol (p = 0.02) and LDL-C (p = 0.03). No other pre/post intervention effects noted (p > 0.05).

Oxidative stress and inflammatory related data for Study 1 are presented in Table 9. No significant interactions or main effects were noted (p > 0.05). Table 10 presents percent change data for selected variables of interest in Study 2. The difference between Glavonoid™ and placebo for cholesterol (p = 0.09), triglycerides (p = 0.20), LDL-C (p = 0.19), and DEXA total body fat (p = 0.21) approached statistical significance.

**Table 9:** Oxidative stress and inflammatory related variables for 22 men and women assigned to Glavonoid™ or placebo for eight weeks.

Variable	Glavonoid™ Pre (n = 11)	Glavonoid™ Post (n = 11)	Placebo Pre (n = 11)	Placebo Post (n = 11)
Malondialdehyde (μmol·L^-1^)	0.95 ± 0.14	0.70 ± 0.11	0.67 ± 0.05	0.66 ± 0.08
Hydrogen Peroxide (μmol·L^-1^)	6.86 ± 1.15	5.24 ± 0.82	4.83 ± 0.66	4.11 ± 0.48
Trolox Equivalent Antioxidant Capacity (mmol·L^-1^)	0.68 ± 0.01	0.63 ± 0.03	0.66 ± 0.02	0.68 ± 0.00
C-Reactive Protein (ng·mL^-1^)	3.86 ± 1.35	3.29 ± 0.93	2.90 ± 1.02	2.02 ± 1.11

Values are mean ± SEM.

No condition × pre/post intervention interaction effects noted (p > 0.05).

No condition effects noted (p > 0.05).

No pre/post intervention effects noted (p > 0.05).

**Table 10:** Percent change data for variables of interest for 23 resistance trained men assigned to Glavonoid™ or placebo for eight weeks.

Variable	Glavonoid™ (n = 12)	Placebo (n = 11)	P value
Cholesterol	8.3 ± 3.5	21.0 ± 6.5	0.09
Triglycerides	13.2 ± 14.8	42.5 ± 16.9	0.20
LDL-C	14.0 ± 6.2	29.7 ± 10.3	0.19
Body Weight	2.5 ± 0.6	2.2 ± 0.7	0.78
DEXA Total Body Fat	2.0 ± 3.0	7.3 ± 2.7	0.21
DEXA Trunk Body Fat	3.1 ± 4.0	10.2 ± 4.6	0.26
Total Fat Mass	4.7 ± 3.5	9.8 ± 3.1	0.29
Total Fat Free Mass	2.1 ± 0.5	1.3 ± 0.6	0.32

Values are mean ± SEM.

Dietary intake data are presented in Table 11 (Study 1) and Table 12 (Study 2). For Study 1, a condition effect was noted for vitamin A (p = 0.05). No other significant effects were noted (p > 0.05). For Study 2, a condition effect was noted for kilocalories (p = 0.002), protein (p = 0.02), carbohydrate (p = 0.02), fat (p = 0.002), and saturated fat (p = 0.004). No other significant effects were noted (p > 0.05).

**Table 11:** Dietary intake for 22 men and women assigned to Glavonoid™ or placebo before and during the final week of an eight week intervention.

Variable	Glavonoid™ Pre (n = 11)	Glavonoid™ Post (n = 11)	Placebo Pre (n = 11)	Placebo Post (n = 11)
Kilocalories	2230 ± 213	2045 ± 208	1976 ± 162	2052 ± 180
Protein (g)	96 ± 10	95 ± 11	82 ± 8	84 ± 5
Carbohydrate (g)	279 ± 25	231 ± 22	251 ± 24	263 ± 31
Fiber (g)	21 ± 2	15 ± 1	15 ± 2	15 ± 2
Sugar (g)	95 ± 9	74 ± 9	92 ± 11	98 ± 17
Fat (g)	76 ± 9	75 ± 10	71 ± 5	73 ± 5
Saturated Fat (g)	25 ± 4	25 ± 4	22 ± 2	25 ± 2
Monounsaturated Fat (g)	13 ± 3	15 ± 3	14 ± 1	15 ± 1
Polyunsaturated Fat (g)	7 ± 1	8 ± 2	7 ± 1	7 ± 1
Trans Fat (g)	1 ± 0	1 ± 0	1 ± 0	1 ± 0
Cholesterol (mg)	218 ± 41	329 ± 53	237 ± 24	223 ± 18
Vitamin C (mg)	71 ± 20	55 ± 9	49 ± 8	66 ± 10
Vitamin E (mg)	5 ± 2	6 ± 2	3 ± 0	4 ± 1
Vitamin A (RE)*	502 ± 169	399 ± 95	221 ± 57	256 ± 5313
Selenium (μg)	47 ±	55 ± 10	47 ± 8	46 ± 5

Values are mean ± SEM.

No significant condition × pre/post intervention interaction effects noted (p > 0.05).

* Condition effect noted for vitamin A (p = 0.05). No other condition effects noted (p > 0.05).

No significant pre/post intervention effects noted (p > 0.05).

**Table 12:** Dietary intake for 22 resistance trained men assigned to Glavonoid™ or placebo before and during the final week of an eight week intervention.

Variable	Glavonoid™ Pre (n = 12)	Glavonoid™ Post (n = 12)	Placebo Pre (n = 10)	Placebo Post (n = 10)
Kilocalories*	2233 ± 141	2469 ± 185	3082 ± 295	3252 ± 336
Protein (g)*	117 ± 11	130 ± 12	154 ± 17	170 ± 22
Carbohydrate (g)*	269 ± 22	316 ± 39	372 ± 41	382 ± 42
Fiber (g)	21 ± 2	22 ± 2	22 ± 3	20 ± 2
Sugar (g)	118 ± 20	143 ± 37	157 ± 32	163 ± 20
Fat (g)*	75 ± 9	78 ± 6	114 ± 15	110 ± 13
Saturated Fat (g)*	23 ± 3	26 ± 3	38 ± 7	38 ± 6
Monounsaturated Fat (g)	16 ± 5	14 ± 2	17 ± 4	20 ± 4
Polyunsaturated Fat (g)	7 ± 2	5 ± 1	7 ± 2	7 ± 1
Trans Fat (g)	1 ± 0	1 ± 1	1 ± 0	1 ± 0
Cholesterol (mg)	477 ± 72	404 ± 61	478 ± 76	541 ± 95
Vitamin C (mg)	111 ± 18	103 ± 19	73 ± 11	93 ± 16
Vitamin E (mg)	11 ± 2	10 ± 2	10 ± 2	17 ± 5
Vitamin A (RE)	410 ± 115	338 ± 86	307 ± 59	472 ± 104
Selenium (μg)	77 ± 17	74 ± 13	70 ± 12	104 ± 23

Values are mean ± SEM.

No significant condition × pre/post intervention interaction effects noted (p > 0.05).

* Condition effect noted for kilocalories (p = 0.002), protein (p = 0.02), carbohydrate (p = 0.02), fat (p = 0.002), and saturated fat (p = 0.004). No other condition effects noted (p > 0.05).

No significant pre/post intervention effects noted (p > 0.05).

## Discussion

The findings from our dual investigation of the effect of the dietary supplement Glavonoid™ indicate the following: In a sample of overweight to grade I-II obese men and women or a sample of athletic men consuming a daily supplemental meal, Glavonoid™ treatment at a dosage of 300 mg·day^-1 ^over a period of eight weeks does not result in any statistically significant difference in anthropometric or biochemical markers of health and adiposity as compared to a placebo. However, when investigating percent change from baseline data (pre intervention to post intervention) in Study 2, subjects in the Glavonoid™ condition did experience less overall fat gain, as well as attenuation in the elevation in selected blood lipids (e.g., cholesterol, LDL-C, and triglycerides; Table 10). While these differences failed to reach statistical significance, it is possible that Glavonoid™ supplementation may provide some benefit to certain individuals engaged in a period of overfeeding. The supplemental meal used in the present study consisted of approximately 30% fat, 20% protein, and 50% carbohydrate, providing subjects an additional 8 kilocalories per kilogram of body mass, resulting in a mean weight gain of only ~2 kilograms. It is possible that Glavonoid™ supplementation could provide greater benefit to individuals regularly consuming higher amounts of dietary fat, or to individuals who routinely overfeed on kilocalorie loads in excess of what was provided in the present study. Of course, additional investigation is needed to confirm such a hypothesis.

Prior animal studies have noted that LFO has the capability of reducing abdominal fat when animals are subjected to high fat feedings [4,11]. Nakagawa and coworkers studied the effect of licorice flavonoids on both abdominal fat and blood glucose regulation in obese female diabetic KK-A^y ^mice and noted attenuated fat/weight gain and increased blood glucose levels compared to the control animals [2]. Collective, although few studies are available in regards to LFO, the animal literature does support the role of LFO in attenuating abdominal adiposity. Of course, the controlled nature of such experiments allows for low variability in response between animals, increasing the chances of detecting statistically significant findings. Coupled with the fact that the animals used in the above studies were exclusively obese (as compared to subjects in Study 1 who were mostly overweight or grade I obese), confounding factors such as dietary and physical activity (which are controlled in animal experiments but cannot be totally controlled in human studies) may account for some of the discrepancies between our findings and those of the animal studies.

That being said, there are at least two human trials using LFO that have noted significant findings in regards to decreasing adiposity. For example, Tominaga et al. noted a significant decrease (~1 kg) in total body fat mass when overweight subjects were supplemented LFO at a daily dosage of 300 mg, 600 mg, or 900 mg for 8 weeks [3]. These data are conflicted with those presented herein, and might be partly explained by the fact that subjects in the Tominaga et al. study were not involved in an exercise program during the course of the study, compared to subjects in the present investigation who were all active. It is possible that subjects in the present study may have altered their activity habits during the course of their participation, confounding the effect of the assigned condition. Unfortunately, when working with human subjects in a free-living environment, such possibilities exist. Tominaga and colleagues performed a similar investigation, noting significant reductions in body mass, attributed to body fat mass, when subjects ingested 300 mg/day of LFO compared to a placebo for 12 weeks [10]. Finally, one other investigation using LFO noted increased resistance of LDL to atherogenic modifications, decreased plasma lipid levels, and decreased systolic blood pressure in patients with hypercholesterolemia [12].

In relation to the above, while we failed to note any statistically significant change in serum lipids following Glavonoid™ treatment, our subjects did not have diagnosed hypercholesterolemia. It is possible that differing results would have been noted in the present study if we had purposely recruited individuals with elevated blood lipids. Moreover, our subjects were all young and healthy, and regularly performed structured exercise. This was not the case in the other trials, as subjects in the Tominaga and co-workers studies consisted of both men and women (post menopausal in one study), aged 24-64 years [3]. Finally, as our sample size was relatively small compared to the other human trials, including additional subjects would have increased our statistical power in detecting an effect in our chosen outcome measures.

It is worth noting that the Glavonoid™ treatment was well-tolerated by subjects and did not result in any adverse outcomes as related to the measured outcome parameters. These findings confirm previous reports in both animals [13,14] and man [9,10] indicating no adverse outcomes with chronic ingestion of LFO.

## Conclusion

In conclusion, our findings do not support prior data pertaining to LFO supplementation in animal and human subjects, as we did not detect any changes of statistical significance with Glavonoid™ treatment at a dosage of 300 mg·day^-1 ^over a period of eight weeks. It is possible that a higher dosage or longer duration of treatment of Glavonoid™ supplementation could provide benefit to individuals, or that the effects of Glavonoid™ may be more pronounced in a homogenous sample of grade II and grade III obese individuals. Moreover, the use of difference assessment tools may allow for more precision in testing certain outcome measures (e.g., CT scans vs. DEXA scans). Additional investigation is needed to confirm such hypotheses. Finally, the Glavonoid™ treatment was well-tolerated by subjects and did not result in any adverse outcomes as related to the measured outcome parameters.

## Competing interests

RJB has received research funding or acted as consultant to nutraceutical and dietary supplement companies. Other authors declare no competing interests.

## Authors' contributions

ZWB and REC were responsible for data collection/entry and assistance with manuscript preparation. RJB was responsible for the study design, overseeing data collection, biochemical work, statistical analysis, and preparation of the manuscript. All authors read and approved the final manuscript.
